# Exploring determinants of community pharmacist-led influenza vaccination in a Middle Eastern country: a national web-based cross-sectional study

**DOI:** 10.1186/s40545-021-00367-y

**Published:** 2021-09-20

**Authors:** Dalal Youssef, Linda Abou-Abbas, Hamad Hassan

**Affiliations:** 1 Preventive Medicine Department, Ministry of Public Health, Beirut, Lebanon; 2 Neuroscience Research Center, Faculty of Medical Sciences, Lebanese University, Beirut, Lebanon; 3 Present Address: Epidemiological Surveillance Unit, Ministry of Public Health, Beirut, Lebanon; 4 Ministry of Public Health, Beirut, Lebanon

**Keywords:** Community pharmacists, Willingness, Influenza vaccine, Enablers, Barriers, Lebanon

## Abstract

**Background:**

Utilizing community pharmacists (CPs) as immunizers has being adopted in various countries as approach to boost influenza vaccination coverage. Our study aims to explore the Lebanese CPs’ willingness to administer influenza vaccine, and to identify factors associated with this willingness.

**Methods:**

This is a web-based, cross-sectional study, conducted over 2 months, from the 1st of November to the end of December 2020. Self-reported data were collected electronically from Lebanese CPs through an anonymous, questionnaire using google form. The collected data were analyzed using the statistical software SPSS (Statistical Package for Social Sciences). Bivariate and multivariable analyses were performed to examine factors associated with the willingness of CPs to administer influenza vaccine.

**Results:**

A total of 412 CPs participated in this survey of which 76.9% are willing to administer influenza vaccines. More than 90% of them had a good overall knowledge score and 88.8% of CPs showed a positive overall attitude score, particularly towards involvement of CPs in influenza vaccine provision. Their willingness to administer vaccine was positively associated with the younger age (aOR = 3.12 with 95% CI (1.597–4.040)), higher education level (aOR = 2.02 with 95% CI (1.093–3.741)), previous experience in immunization (aOR = 2.72 with 95% CI (1.320–5.627)) and urbanicity of pharmacy (aOR = 1.542 with 95% CI (1.219–4.627)). Extensive working hours (aOR = 2.34 with 95% CI (1.131–4.845)), working in pharmacies that are operating round-the-clock, showing positive attitude towards immunization (aOR = 3.01 with 95% CI (1.872–6.422)) and towards provision of influenza vaccines (aOR = 13.72 with 95% CI (13.721–38.507)) were also positively associated to this willingness. Conversely, patient privacy (aOR = 0.55 with 95% CI (0.079–0.983)), time and cost for professional development (aOR = 0.55 with 95% CI (0.172–0.918)), limited patient’s trust (aOR = 0.39 with 95% CI (0.203–0.784)), financial remuneration (aOR = 0.18 with 95% CI (0.088–0.377)), and requirement of formal certification in vaccine administration (aOR = 0.07 with 95% CI (0.020–0.279)) were negatively associated to this willingness.

**Conclusion:**

Addressing the unearthed concerns related to utilizing CPs as influenza immunizers through a concerted effort is a key to success in any future implementation of vaccination services in pharmacies.

## Background

Influenza is a substantial cause of morbidity and mortality worldwide, with 291,243 to 645,832 estimated seasonal influenza-associated respiratory deaths occurring yearly [1]. It also increases hospital admissions particularly during the winter season, impacting work productivity and leading to considerable economic loss [2].

Given that vaccination is considered the mainstay and the most effective control measure for influenza and its complications[3, 4], the importance of increasing the vaccination rate in the general population and particularly in high-risk groups consequently, reducing the number of influenza cases and a subsequent lessening in healthcare use and costs. Since the launch of the Global Action Plan for Influenza Vaccines (GAP) by the World Health Organization (WHO) in 2006, and despite the considerable increase of global production capacity for influenza vaccine, the amount needed to achieve the goal of equitably immunizing 70% of the world’s population is still unreachable, and further interventions are required [5].

Recognizing its geographic location and large population, the Eastern Mediterranean Region (EMR), is affected seasonally with influenza. However, there is a scarcity of data on seasonal influenza vaccine coverage in the region [6]. According to a review of seasonal influenza in the EMR, alarming low influenza vaccination coverage was reported, emphasizing the development of vaccination policies [7]. Since many countries are currently struggling with low influenza vaccination coverage and rarely met the target immunization coverage, it calls for the question of what other countries are doing and managing to improve their vaccination rates and to approach or reach the vaccination coverage target in the general population and the most vulnerable patients [8]. In this context, we found that some countries such as United Kingdom (UK) and the Netherlands have recognized the benefits of involving additional vaccine providers such as community pharmacies in supporting flu vaccination services. In recent decades, several countries have expanded the role of pharmacists to provide influenza vaccinations [8]. Besides, community pharmacy-delivered flu vaccination is now perceived as an integral part of the health system and recognized for its benefits in many countries such as Canada, England. The literature showed that the influenza vaccination uptake in England in areas where flu vaccinations were provided by community pharmacies was higher than in four similar areas that did not provide the service.

Lebanon is a small country in the Middle East with a population of around 6.7 million people including refugees and 4185 registered community pharmacies with the Order of Pharmacists (LOP). Community pharmacies are privately owned on a for-profit basis and are the only legal provider of prescription and non-prescription drugs to the Lebanese community. However, according to the law, vaccines cannot be administered at these facilities. Annually, the Lebanese Ministry of Public Health (MOPH) issued a circular recommending the influenza vaccination for people with an increased risk of complications including elderly people, pregnant women, and children less than 5 years old, persons with certain chronic diseases such as those with asthma, chronic heart or lung conditions, or who are immunocompromised, and health care workers. Nonetheless, a cross-sectional survey in Lebanon found that the overall 2014–2015 seasonal influenza vaccination rate among ambulatory adults was 27.6%. The sample population included participants in high-risk groups where the immunization rates ranged from 18.2 to 35% [9] which is considered alarming.

Presently, CPs in Lebanon are not permitted by law to administer vaccines. However, it is well known that CPs are actively involved in public education. According to our knowledge, there are no studies or clear understanding on community pharmacist’s willingness to become an influenza provider in Lebanon. In the light of the current situation, it is of great interest, to explore, prior to embarking on a program expanding the scope of pharmacy practice to include the provision of influenza immunization services, the desire of CPs to administer influenza vaccines, and boosting the influenza vaccination coverage among the Lebanon population. Such information may inform policy development and statutory reform around implementing pharmacy-led influenza vaccination service.

The objectives of this study are to assess the Lebanese CP’s willingness to become an influenza immunizer, their knowledge and attitudes toward immunization as well and to identify the enablers and barriers associated with this willingness.

## Methods

### Study design and population

This is a web-based, cross-sectional study, conducted during a period of 8 weeks from the 1st of November to the end of December 2020. It was carried out, using a random sampling of Lebanese community pharmacies. Randomization was performed on provincial level to obtain a representative sample of community pharmacies across Lebanon. CPs working currently in pharmacy setting and who agreed to participate to the study were eligible for participation. Exclusion criteria included: clinical pharmacists, retired CPs, those who were out of the country at the time of the survey, as well as those not practicing actually. Pharmacists who were unreachable due to change of their contact information during the time of the survey and those who refused to participate in the study were also excluded.

The list of community pharmacies was provided by the Lebanese Order of Pharmacists (LOP), and then these pharmacies were categorized per province. The number of CPs chosen from each province is proportional to the population of these pharmacies existing in this province.

### Tool development

A 60-item questionnaire was developed and designed specifically by the authors to assess the community pharmacists’ willingness, to administer influenza vaccines at different Lebanese provinces, and to determine the factors associated with their willingness. The questions used were adopted from similar studies done in other countries [10]. A panel of experts was asked to comment on clarity, wording, interpretability and relevance of the items in the survey. Given that Arabic is the native language in Lebanon, an Arabic version of the questionnaire was made based on standard translation guidelines [11]. The questionnaire was pre-tested among 30 community pharmacists for survey flow, functionality, readability, and clarity. Minor amendments were made based on the feedback of the pre-test. The average time for filling the survey was 12 min. The questionnaire was self-administered and consisted of closed-ended questions. It was divided into multiple parts:

The first part includes the patient’s sociodemographic characteristics including age, gender, marital status, profile, level of education, clinical experience and working hours. Participants were also asked about some information related to the community pharmacy where they work (urbanicity, province, opening hours, etc.).

The second part consists of community pharmacists’ willingness to expand their practice scope to include influenza vaccine provision. Question was answered on a yes/no basis.

The third part is about knowledge of pharmacists regarding influenza vaccine. All the items were answered on a true/false basis and an additional “do not know” option. A correct response had a value of ‘1′ and a "wrong" or don’t know response had a value of ‘0′. Hence, the cumulative score for all 26 knowledge questions would range from 0 to 26 points. Participants ‘overall knowledge was categorized using modified Bloom’s cut-off point, as good if the score was between 60 and 100% (16–26 points), and poor if the score was less than 60% (< 16 points).

The fourth part is about pharmacists’ attitudes towards vaccination and utilizing pharmacists as immunizers, elements needed to incorporate influenza immunization in pharmacists’ practice scope and barriers perceived by pharmacists for the pharmacist-run of influenza vaccination service. The fourth part included a 3-points Likert scale to assess further the extent to which patients agreed. For the items describing the attitudes, an agree/strongly agree response had a value of ‘1′ and a "disagree", “strongly disagree", or neutral response had a value of ‘0′. Hence, the cumulative score for all 13 attitudes questions would range from 0 to 13 points. Participants ‘overall attitude” was categorized using modified Bloom’s cut-off point, as good if the score was between 60 and 100% (8–13 points), and poor if the score was less than 60% (< 13 points). To note, a reverse coding was performed to the attitude item number 4 (A4: considering natural infection or a healthy lifestyle is effective alternatives to vaccines). Similar method was used for the dichotomization of attitudes subscales.

### Sample size calculation

Based on a total population size of 4185 community pharmacies registered with OPL, a 95% confidence level and an absolute error of 5%, a minimal sample of 352 pharmacists was required using Raosoft sample size calculator to allow adequate power for bivariate and multivariable analyses.

### Data collection

As the Lebanese government recommended the public to minimize face-to-face interaction, CPs from different provinces were electronically invited to participate in confidence, no personal identifiers were collected and no incentives offered. CPs were contacted firstly via phone call and notified about the survey and its purpose. Their consent to participating was requested and then the link of the anonymous online questionnaire (Arabic and English versions) using a Google form was sent to only those who consented, with a request to not forward it further. Forwarding of the link to inappropriate groups or persons may be prevented to some extent by mentioning the inclusion and exclusion criteria in the request message. Response rates to this online survey was increased since data collector preceded by a phone call to potential respondents. This link of the study included a brief introduction to the background, the objective of the survey, and instructions for filling the questionnaire.

### Ethical considerations

An oral and written informed consent was obtained for each participant. They were reassured that their participation is voluntary and that they were free to withdraw at any time. In addition, all information were gathered anonymously and handled confidentially. The study design assured adequate protection of study participants, and neither included clinical data about patients nor configured itself as a clinical trial. Hence, this study was exempted from ethical approval of the ministry of Public Health.

### Statistical analysis

The collected data were exported to a Microsoft Excel 2016 for cleaning and coding. The cleaned data were exported and analyzed using the statistical software SPSS (Statistical Package for Social Sciences), version 22.0. Since the data were collected via a link, no missing values were recorded as all questions were required. Descriptive statistics were reported using frequency with percentages for categorical variables. To check scale reliability, Cronbach’s alpha was conducted on computed scores. Bivariate and multivariable analyses were performed to examine factors associated with the dependent variable (willingness to administer influenza vaccine). The relation between nominal variables was tested using the Chi-squared test. The variables in bivariate analysis with *p*-value < 0.2 were entered into multivariable logistic regression. Adjusted odds ratio and their 95% confidence intervals were reported. The final logistic regression model to determine the predictors for willingness to vaccinate was reached after confirming the adequacy of the data using the Hosmer and Lemeshow test. The level of statistical significance was set at a *p*-value < 0.05.

## Results

### Baseline information

**Fig. 1:**
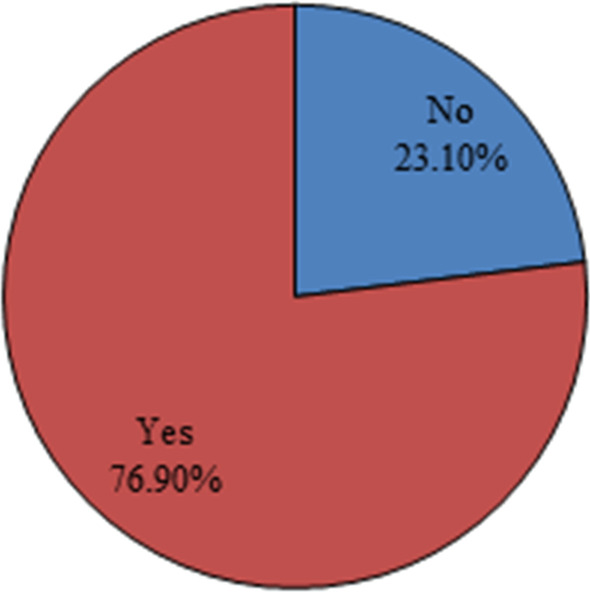
Pharmacist's willingness to provide influenza vaccine

A total of 412 community pharmacists participated in this survey of which 45.1% were males. Table 1 summarizes the baseline characteristics of the participants. The majority of respondents (62.4%) were aged less than 40 years old. Around half of them (44.4%) had an educational level higher than a pharmacy diploma degree. With respect to their experience in immunization, only 23.3% reported previous experience in immunization. Most of pharmacists worked in pharmacies located in urban area (68%). Out of all, 76.9% of the surveyed community pharmacists were willing to expand their practice scope to include provision of influenza vaccines in their pharmacies (Fig. 1).

**Table 1:** Baseline characteristics of the study participants (*N* = 412)

	*n*	%
Gender		
Male	186	45.10
Female	226	54.90
Age (years)		
Less than 40 years old	257	62.40
40 years old and above	155	37.70
Educational level		
BS pharmacy	229	55.60
More than BS degree	183	44.50
Years of experience		
0–5 years	137	33.30
6–10 years	95	23.10
More than 10 years	180	43.70
Previous experience in immunization		
No	96	23.30
Yes	316	76.70
Pharmacist's working hours per week		
24 h or less	93	22.60
More than 24 h	319	77.40
Geographic location of the pharmacy		
Rural	132	32
Urban	280	68
Number of hours/week pharmacy is open		
Less than 80 h	153	37.2
80–120 h	228	55.30
7 days 24/24	31	7.50

*n* frequency, *%* percentage, *BS* Bachelor of Science, more than BS degree included PharmD, Master, PhD…

### Knowledge

**Fig. 2:**
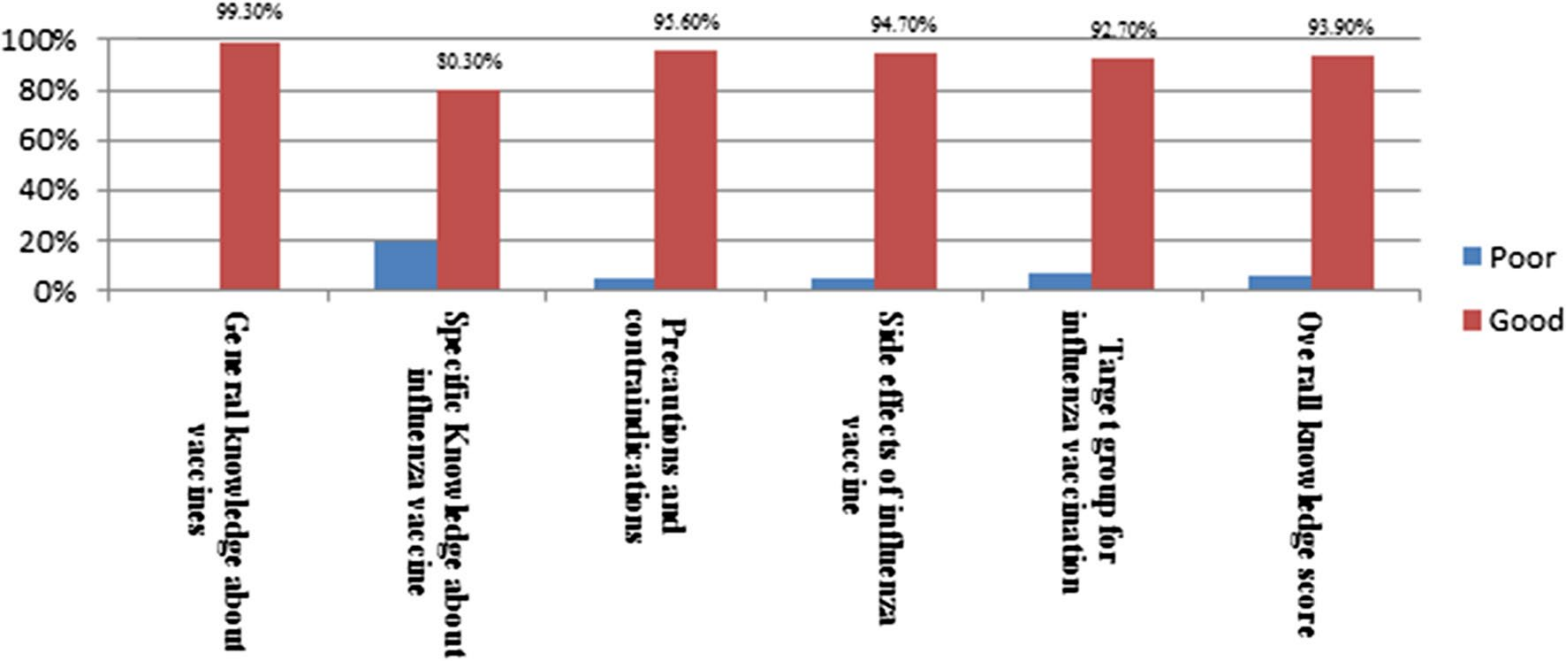
Pharmacist's knowledge domains

More than 90% of community pharmacists had a good overall knowledge score. They were ranked knowledgeable in all knowledge domains: general knowledge about vaccines (99.3%), specific knowledge about influenza vaccine (80.3%), side effects of the vaccine (94.7%), precautions and contraindications (94.7%) and target groups for influenza vaccine (92.7%) (Figs. 1, 2).

Table 2 displays the community pharmacists’ answers to influenza vaccine knowledge items. The bulk of CPs answered correctly to the knowledge questions. However, only 49% of surveyed pharmacists were aware that the quadrivalent flu vaccines protect against four different flu viruses: H1N1, H3N2, and two influenza B viruses. Similarly, only 45.4% of them were aware that people who get a seasonal flu vaccine can still get sick with flu symptoms and 68.7% were familiar that influenza vaccine, like other injections, can occasionally cause fainting.

**Table 2:** Community pharmacists responses to influenza vaccine knowledge items

		Incorrect	Correct	I don’t know
#		*n* (%)	*n* (%)	*n* (%)
Domain 1: General knowledge about vaccines				
K1	Vaccines are critical to the prevention and control of infectious diseases outbreaks	3 (0.7%)	409 (99.3%)	0 (0%)
K2	The ingredients of the vaccine include: the antigen, adjuvants, preservatives, and stabilizers	6 (1.5%)	375 (91%)	31 (7.5%)
K3	Vaccines are safe and serious problems from the vaccine are very rare	20 (4.9%)	383 (93%)	9 (2.2%)
K4	Every vaccine must go through extensive and rigorous testing before it can be introduced	6 (1.5%)	402 (97.6%)	4 (1%)
K5	Following the introduction of a vaccine, close monitoring continues to detect any unexpected adverse side effects and assess the effectiveness	22 (5.3%)	377 (91.5%)	13 (3.2%)
Domain 2: Specific Knowledge about influenza vaccine				
K6	An annual seasonal flu vaccine is the best way to help protect against flu	38 (9.2%)	358 (86.9%)	16 (3.9%)
K7	Flu vaccines cause antibodies to develop in the body about two weeks after vaccination and provide vaccinations	8 (1.9%)	404 (98.1%)	0 (0%)
K8	Quadrivalent flu vaccines protect against four different flu viruses: an influenza A (H1N1) virus, an influenza A (H3N2) virus, and two influenza B viruses	107 (26%)	202 (49%)	103 (25%)
K9	A trivalent flu shot made using an adjuvant (an ingredient that helps create a stronger immune response), approved for people 65 years of age and older	46 (11.2%)	292 (70.9%)	74 (18%)
K10	Influenza vaccination doesn’t increase the risk of being infected with the SARS-CoV-2 (COVID-19) virus or any other respiratory virus	56 (13.6%)	300 (72.8%)	56 (13.6%)
K11	Flu vaccine should be taken before flu viruses begin spreading in the community	7 (1.7%)	384 (93.2%)	21 (5.1%)
K12	Vaccination could continue to be offered throughout the flu season, even into January or later	25 (6.1%)	342 (83%)	45 (10.9%)
K13	People who get a seasonal flu vaccine and still get sick with flu symptoms	112 (27.2%)	187 (45.4%)	113 (27.4%)
Domain 3: Side effects of the vaccines				
K14	Common side effects from a flu shot include soreness, redness, and/or swelling where the shot was given, headache (low grade), fever, nausea, muscle aches, and fatigue	48 (11.7%)	291 (70.6%)	73 (17.7%)
K15	The risk of an allergic reaction can be decreased by effective screening prior to vaccination	43 (10.4%)	316 (76.7%)	53 (12.9%)
K16	Providers should report any clinically significant adverse event occurring after administration of the vaccine even if they are unsure whether the vaccine caused the event	3 (0.7%)	400 (97.1%)	9 (2.2%)
K17	Flu vaccine side effects are generally mild and go away on their own within a few days	3 (0.7%)	403 (97.8%)	6 (1.5%)
K18	The flu shot, like other injections, can occasionally cause fainting	85 (20.6%)	283 (68.7%)	44 (10.7%)
K19	Life-threatening allergic reactions to flu shots are very rare	10 (2.4%)	386 (93.7%)	16 (3.9%)
K20	Signs of serious allergic reaction can include breathing problems, hoarseness or wheezing, hives, paleness, weakness, a fast heartbeat, or dizziness	23 (5.6%)	318 (77.2%)	71 (17.2%)
Domain 4: Precautions and contraindications				
K21	Anaphylactic reaction to a previous dose of vaccine or a vaccine component is a contraindication to further doses of the same vaccine or to the same component in other vaccines	7 (1.7%)	387 (93.9%)	18 (4.4%)
K22	Multiple sclerosis is a not a contraindication to influenza vaccine	55 (13.3%)	240 (58.3%)	117 (28.4%)
Domain 5: People at risk of influenza vaccination				
K23	Children aged 6 months through 4 years (59 months) should take influenza vaccines	30 (7.3%)	382 (92.7%)	0 (0%)
K24	Pregnant women and people with certain chronic health conditions can get a flu shot	37 (9%)	375 (91%)	0 (0%)
K25	Children younger than 6 months of age are too young to get a flu should not get flu shot	30 (7.3%)	382 (92.7%)	0 (0%)
K26	Annual influenza immunization is recommended for all health care professionals in contact with individuals in high-risk groups	29 (7.1%)	383 (92.9%)	0 (0%)

### CP’s attitudes toward influenza immunization

**Fig. 3:**
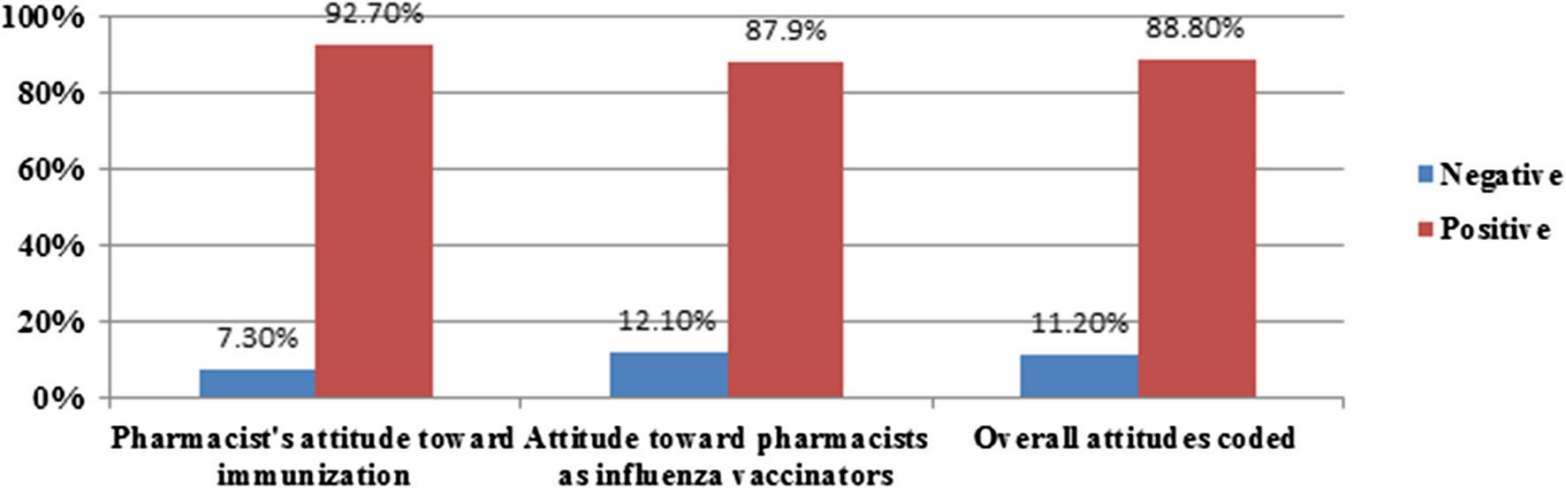
Pharmacist's attitudes towards immunization

In total, 88.8% of CPs showed a positive overall attitude score. More than 90% have a positive attitude towards vaccination and 87.9% perceived positively the involvement of CPs in influenza vaccine provision (Fig. 3).

Table 3 displays CP’s level of agreement towards immunization. The majority of pharmacists have agreed on the importance of immunization and that vaccine more health benefits than health risks. Only 20.4% of them agreed that natural infection or a healthy lifestyle is effective alternatives to vaccines and less than half of them considered that the provision of immunizations to adults, as it is currently done, is adequate. Concerning their attitudes towards utilizing pharmacist as immunizer, the majority of them (91.4%) thought that pharmacists be permitted to expand their practice to include the administration of adult vaccines. Similarly, 95.2% of them considered that CPs can play an important role in advertising and promoting vaccination in addition to the increase of vaccination coverage rate among adults rate. Despite that only 51% of them considered that CPs have received adequate teaching/training about influenza vaccine administration during their pharmacy training, 73.5% of them thought that CPs have good knowledge of influenza vaccines’ indications and contraindications. On other hand, 87.3% of them deliberated that allowing pharmacists to vaccinate can reduce costs of influenza vaccination paid by patients.

**Table 3:** Community pharmacists attitudes items towards immunization

		Strongly disagree/disagree	Neutral	Strongly agree/agree
		*n* (%)	*n* (%)	*n* (%)
Attitudes towards immunization				
A1	I think that the provision of influenza immunization to adults, as it is currently done, is adequate	167 (40.5%)	56 (13.6%)	189 (45.9%)
A2	I think it is important that adults receive all vaccines recommended by Health authorities including influenza vaccine	7 (1.7%)	22 (5.3%)	383 (92.9%)
A3	I think that increasing the proportion of the population including adults who receive recommended immunizations (flu shot) is important	14 (3.4%)	31 (7.5%)	367 (89.1%)
A4	I think that natural infection or a healthy lifestyle is effective alternatives to vaccines. (R)	252 (61.2%)	76 (18.4%)	84 (20.4%)
A5	I think that vaccines produce more health benefits than health risks	22 (5.4%)	15 (3.6%)	375 (90.1%)
Attitudes towards providing influenza vaccination by pharmacists				
A6	I think pharmacists have good knowledge of influenza vaccines and their indications and contraindications	39 (9.5%)	70 (17%)	303 (73.5%)
A7	I think that community pharmacists received adequate teaching/training about influenza vaccine administration during their pharmacy training	126 (30.6%)	76 (18.4%)	210 (51.0%)
A8	I think community pharmacists are easily accessible to the community (hours of availability, geographical distribution….) especially to adults to administer recommended vaccines	193 (46.8%)	16 (3.9%)	371 (90.1%)
A9	I think that providing vaccination through community pharmacy will improve the overall rate of vaccination among adults and especially in elderly	21 (5.1%)	29 (7%)	362 (87.8%)
A10	If pharmacists were permitted to immunize, most adults would feel comfortable receiving their recommended vaccinations from a pharmacist	21 (5.1%)	47 (11.4%)	244 (82.6%)
A11	I think community pharmacists can play an important role in advertising and promoting vaccination	12 (2.9%)	7 (1.7%)	393 (95.2%)
A12	I think that pharmacists should be permitted to expand their practice to include the administration of recommended adult vaccines	28 (6.8%)	35 (8.5%)	349 (91.4%)
A13	I think that allowing pharmacists to vaccinate can reduce costs of vaccination paid by patients which would be the main determining factor of "if patients will choose to receive a vaccine from a pharmacist"	3 (0.7%)	34 (8.3%)	323 (87.3%)

**Fig. 4:**
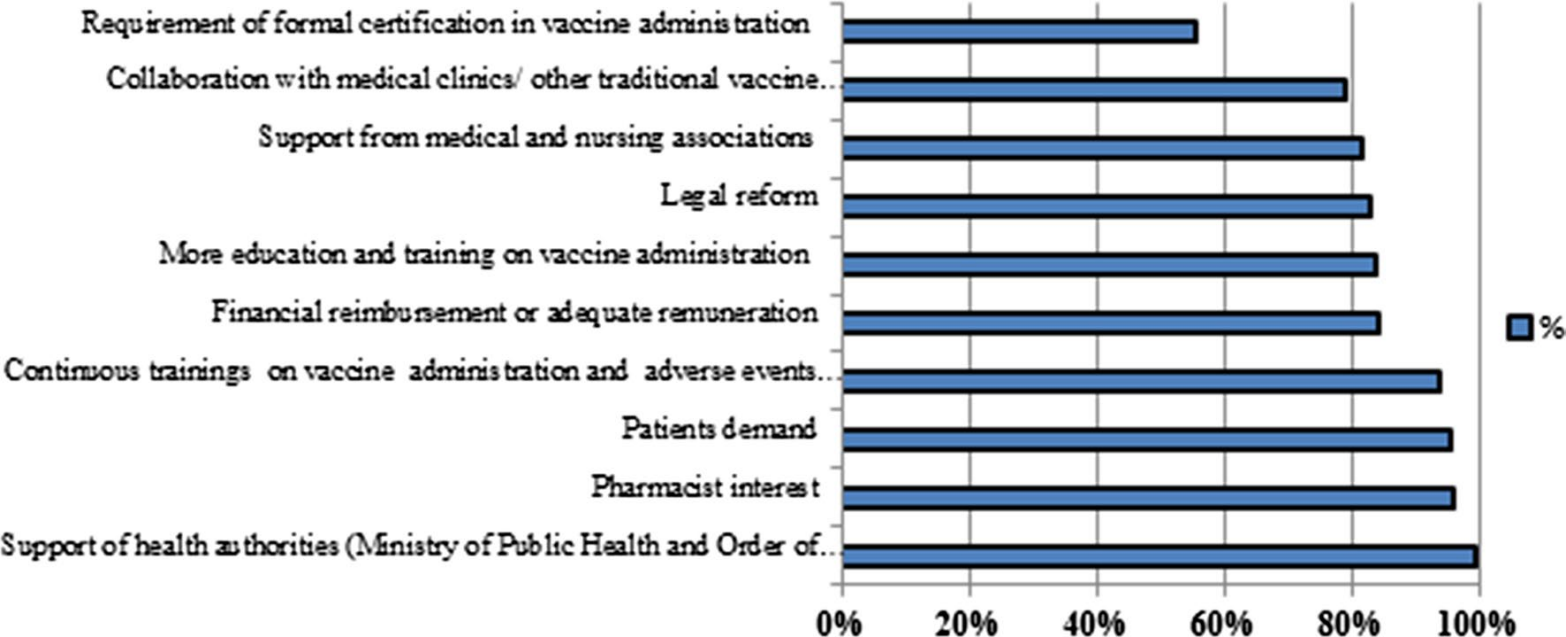
Elements needed for implementing influenza immunization services in pharmacies

The main elements required for implementing influenza immunization services as perceived by CPs were support from health authorities, pharmacist interest, patient demand, continuous training on vaccine administration and management of adverse events and adequate remuneration (Fig. 4).

Table 4 summarizes the barriers for implementing influenza vaccination in pharmacies as perceived by CPs. The top ranking barriers were conflicts with other professionals who are eligible to vaccinate (75%), liability and malpractice concerns (54.6%), remuneration (49%), time and cost needed for professional development and training (44.4%) and CP’s lack of knowledge of how to manage adverse events after immunizing (41.3%). However, insufficient human resources (20.9%) and lack of space (11.9%) were ranked the least.

**Table 4:** Concerns and barriers perceived by pharmacists about providing influenza vaccine in pharmacies

	Disagree/neutral	Agree
	*n* (%)	*n* (%)
Conflicts with other professionals who are eligible to vaccinate	103 (25%)	309 (75%)
Liability and malpractice concerns	187 (45.4%)	225 (54.6%)
Remuneration concerns	210 (51%)	202 (49%)
Time and cost needed for professional development and training	229 (55.6%)	183 (44.4%)
Lack of knowledge of adverse events after immunizing and how to manage such situation	242 (58.7%)	170 (41.3%)
Pharmacists are less trusted by patients to provide such service	244 (59.2%)	168 (40.8%)
Lack of knowledge of vaccine indications, contraindications and safely administration	259 (62.9%)	153 (37.1%)
Costs associated with professional development and training	277 (67.2%)	135 (32.8%)
Patient privacy is an issue with regard to administering immunization in a community pharmacy	284 (68.9%)	128 (31.1%)
Insufficient staff or resources to implement	326 (79.1%)	86 (20.9%)
Lack of pharmacy space and facilities to store and to administer vaccine	363 (88.1%)	49 (11.9%)

*n* frequency, *%* percentage

### Factors associated with the willingness of pharmacists to vaccinate under any circumstances

Table 5 represents the multivariable logistic regression of the factors associated with pharmacists’ willingness to administrate influenza vaccines in their settings. Our results showed that the willingness was positively associated with the younger age, where the willingness to immunize among pharmacists aged 40 years old or less was 3.11 times higher than their counterparts aged more than 40 years (aOR = 3.11 with 95% CI (1.597–4.040)). Similarly, CPs having an education level higher than diploma in pharmacy were 2 times more likely to administer influenza vaccine (aOR = 2.02 with 95% CI (1.093–3.741)) than their colleagues with BS degree. Previous experience in immunization (aOR = 2.72 with 95% CI (1.320–5.627)) and urban location of pharmacy (aOR = 1.542 with 95% CI (1.219–4.627)) were positively associated to pharmacists willingness to vaccinate comparing to those who lack from a previous experience in immunization and working in rural areas. Concerning working hours, pharmacists working more than 24 h per week were 2.3 times (aOR = 2.341 with 95% CI (1.131–4.845)) more willing to provide influenza vaccine. Likewise, respondents working in pharmacies that are operating round-the-clock were 7.9 times more willing to be involved in influenza vaccination comparing to their colleagues working in pharmacies operating less than 50 h per week.

**Table 5:** Multivariable logistic regression of the factors associated with the pharmacist’s willingness to administer influenza vaccine in their pharmacies

	Willingness to administer influenza vaccines					
	No	Yes	*P*-value	aOR	95% C.I. for aOR	
	*n* (%)	*n* (%)			Lower	Upper
Age			0.002			
More than 40 years old	37 (28.7%)	92 (71.3%)	1			
40 years old or less	53 (20.6%)	204 (79.4%)	0.001	3.106	1.597	6.040
Education level			0.012			
BS pharmacy	60 (26.2%)	169 (73.8%)	1			
More than BS degree (PharmD, Master, PhD…)	36 (19.7%)	147 (80.3%)	0.025	2.022	1.093	3.741
Previous experience in immunization			0.043			
No	31 (32.30%)	65 (67.7%)	1			
Yes	65 (20.6%)	251 (79.4%)	0.007	2.725	1.320	5.627
Geographical location			0.041			
Rural	38 (28.8%)	94 (71.2%)				
Urban	58 (20.7%)	222 (79.3%)	0.039	1.542	1.219	4.627
Number of hours pharmacist is working per week			0.006			
0–24 h	35 (37.6%)	58 (62.4%)	1			
More than 24 h	61 (19.1%)	258 (80.9%)	0.022	2.341	1.131	4.845
Number of hours pharmacy is open per week			0.047			
Less than 50 h	6 (33.3%)	12 (66.7%)	1			
50–120 h	87 (24%)	276 (76%)	0.259	2.157	0.568	8.199
7 days 24/24	3 (9.7%)	28 (90.3%)	0.029	7.993	1.238	11.604
Attitude towards immunization			0.006			
Negative	20 (66.7%)	10 (33.3%)	1			
Positive	75 (19.6%)	307 (80.4%)	0.041	3.014	1.872	10.422
Attitudes towards provision of influenza vaccine by community pharmacists			< 0.001			
Negative	30 (60%)	20 (40%)	1			
Positive	65 (18%)	297 (82%)	< 0.001	13.721	4.889	38.507
Patient privacy concern			0.01			
No	57 (20.1%)	227 (79.9%)	1			
Yes	38 (29.7%)	90 (70.3%)	0.011	0.553	0.079	0.983
Time and cost needed for professional development and training			0.01			
No	54 (23.6%)	175 (76.4%)	1			
Yes	41 (22.4%)	142 (77.6%)	0.019	0.551	0.172	0.918
Pharmacists are less trusted by patients to provide such service			0.012			
No	64 (26.2%)	180 (73.8%)	1			
Yes	31 (18.5%)	137 (81.5%)	0.008	0.399	0.203	0.784
Financial reimbursement or adequate remuneration concern			< 0.001			
No	9 (13.6%)	57 (86.4%)	1			
Yes	86 (24.9%)	260 (75.1%)	< 0.001	0.182	0.088	0.377
Requirement of formal certification in vaccine administration			< 0.001			
No	22 (12%)	161 (88%)	1			
Yes	73 (31.9%)	156 (68.1%)	< 0.001	0.075	0.020	0.279

*n* frequency, *%* percentage, *aOR* adjusted odds ratio, *CI* confidence interval, *P*-value < 0.05 is considered statistically significant

Concerning their attitudes, community pharmacists showing positive attitude towards immunization (aOR = 3.01 with 95% CI (1.872 to 6.422)) and towards provision of influenza vaccines by pharmacists (aOR = 6.15 with 95% CI (4.889 to 8.507)) were more willing to administer influenza vaccine compared to their colleagues who expressed negative attitudes.

To note, small samples among subgroups could produces odds ratio that are too large (Nemes, Jonasson, Genell, & Steineck, 2009). Odds ratios tend to be farther away from 1.0 (higher for positive relationships, lower for negative relationship) for smaller samples. On the contrary, community pharmacists who considered patient privacy (aOR = 0.55 with 95% CI (0.079 to 0.983)), time and cost needed for professional development and training (aOR = 0.55 with 95% CI (0.172 to 0.918)), lack of patient’s trust towards pharmacists (aOR = 0.39 with 95% CI (0.203–0.784)), adequate financial remuneration (aOR = 0.18 with 95% CI (0.088–0.377)), and requirement of formal certification in vaccine administration (aOR = 0.07 with 95% CI (0.020–0.279)) concerns were negatively associated to the willingness to administer influenza vaccine compared to their counterparts who did not perceive such concerns.

## Discussion

Immunization is a cornerstone of public health through the prevention of infectious diseases and their complications including influenza. One approach used to boost influenza vaccination coverage, has been to expand the scope of practice of CPs beyond dispensing medications towards providing vaccination [8]. This nationwide study is the first known in Lebanon to assess community pharmacists’ willingness to provide influenza vaccine in their pharmacies. It adds to current literature toward vaccinations by exploring the extremely important topic of the knowledge, attitudes, and perceived barriers for the instigation of such process among an appropriate and accurate representation of community pharmacists in Lebanon. It also provides important insights about factors associated with this willingness.

The main findings in our study were that more than three-quarters of the surveyed CPs were willing to administer influenza vaccines in their pharmacies. Compared to a study conducted among Canadian CPs, only 51% of them reported willingness to incorporate provision of immunization into their personal practice [10]. Hence, Lebanese CPs showed a strong engagement and interest in expanding their practice scope to include provision of influenza vaccine, which is considered a boosting foundation required to the embarkation of such process.

With regard to CP’s knowledge, the bulk of them owned an overall good knowledge score. They were aware and cognizant in all knowledge domains: general and specific knowledge about the vaccine, side effects, precautions and contraindications and target groups. Our results were inconsistent with the findings of a study conducted in Jordan where pharmacists lack the knowledge of how to carry out a proper assessment before administering the shot or how to handle any adverse reactions after administration and do not have enough knowledge of proper needle disposal [12]. Since good knowledge about influenza vaccine is crucial for CPs to prepare them to provide adequate education to the public and, subsequently, improving their performance and self-reliance in administrating vaccines.

Concerning attitudes, it is notable that this study demonstrated that the bulk of CPs had a positive overall attitude score. They showed a highest perception of the benefits of influenza vaccination as they consider it more beneficial than risky. They also highlighted the importance of vaccination and refused to consider that natural infection or a healthy lifestyle as effective alternatives to vaccines. Additionally, most respondents rated the importance of their role in advertising and promoting vaccination in addition to the increase of vaccination coverage rate among adults rate, likewise, CPs also believed that they should be permitted to expand their practice to include the administration of adult vaccines. This is consistent with the results reported in a study conducted in Italy [13]. Furthermore, a study conducted in the United States exhibited that 89.3% of pharmacists thought that vaccines for adults were one of the top priorities in overall patient management and 96.3% considered vaccinations of adults within their scope of practice [14, 15].

The main components cited by CPs as requirements for the implementation of influenza immunization services, included support from health establishments, patient demand, and legislative reform. However, any expansion of CPs practice scope could not be achievable without a statutory allowance and a fully support from health authorities. Patient demand is also considered as a pivotal factor which is linked to the pharmacy image and the patient attitude toward CPs services. Despite that many studies in many countries such as in Europe, USA, and Canada disclosed a positive overall perception of CP services [16–21].

A study conducted in Lebanon revealed a poor public perception and attitude toward CPs in highly qualified and dedicated pharmacists [22] hence, the importance of upgrading a trust-based patient–pharmacist relationship. In addition, there is a need to a multi-stakeholders collaboration (OPL, MOPH, and CPs) to improve public awareness about the role of CP as a healthcare professional and potential immunizer. Indeed, there is a need to assess the attitudes and the perspectives of the Lebanese community towards pharmacy-led influenza immunization.

Additionally, continuous education on vaccine safely administration and management of adverse events and adequate remuneration were also underscored by CPs among needed components. This shed light on the importance of formal certification that addresses the safe and effective administration of vaccines as requisite for allowing pharmacists to be immunizers combined with regular trainings. With regard to adequate remuneration, it should be noted that in Lebanon out-of-pocket influenza vaccine expenses are paid by the citizens, including the vulnerable groups and that the cost involved in visiting a physician for vaccination has additional cost consideration.. Despite that flu vaccines supplied and administered in a pharmacy setting could offer savings and convenience, an adequate and a standardized remuneration of CPs immunization services is crucial for the sustainability of such services.

With regard to barriers for implementing influenza vaccination in pharmacies, the top ranked barriers were the conflicts with other professionals who are eligible to vaccinate. This issue is commonly reported by many countries where a fierce opposition and lack of support from the physicians toward pharmacist-run immunization was indicated [23]. It was defended by the fact that CPs were not adequately or effectively trained to do vaccination or to manage adverse effects following vaccine administration [24–26].

Also, cost of vaccination services provided at physician clinic will includes consultation fees in addition to influenza vaccine price and provision fees makes it expensive for the patient. Hence, such struggle could be originated from financial conflict of interest. Moreover, liability and malpractice concerns and CP’s lack of knowledge of how to manage adverse events after immunizing were also perceived as main barriers. Generally, low rates of adverse events were associated with flu vaccinations; the incidence of anaphylaxis in any clinical setting ranges from 0.7 to 2 per 1,000,000 doses [27]. Although there are few reports of incidence of side effects in pharmacy specifically, in the first season of instigation of flu vaccination in pharmacy in both Portugal (2008/09) and Ireland (2010/11), no incidences of anaphylaxis were recorded [28, 29].

Despite the low risk of serious adverse event, the need of training for CPs still integrates a vigorous component around safety. Recognizing this fact, countries have been proactive and adopted training programs. For example, the American Pharmacists Association (APhA) have developed the Pharmacy-Based Immunization Delivery Training module including vaccine administration skills as well as addressing injection technique, response to anaphylactic incidents and basic life support [30]. Hence, a nationally standardized approach and rolling out of a recognized and accredited training course are recommended. In addition, there is need to maintain CP’s competency through an ongoing service delivery training and yearly updating of skills. Lastly, recertification courses complying with updated guidelines might be required.

One noteworthy finding is that the remuneration was perceived simultaneously by CPs as main component for the instigation of pharmacy-run immunization and as main concern. The payment for provision of flu vaccination is related to the type of healthcare system. In Lebanon, such service can be provided privately, where the patient pays the provider directly, and will not be reimbursed to the patient.

Our findings showed that the willingness of CP’s to provide influenza vaccine was positively associated with younger age, higher education level (more than diploma in pharmacy), previous experience in immunization and urban location of pharmacy. This could be explained that higher education level could be associated with acquirement of additional knowledge related to vaccinology, therefore making CPs more confident in expanding their role. Similarly, previous experience in immunization could enhance their self-reliance as immunizer, hence increasing their intention to administer vaccine.

Interestingly, young CPs were more likely to administer influenza vaccination than their counterparts who were older and having greater period. This could be understood in the light of the data that showed that Lebanon has a pharmacist-to-population ratio of 20.3 pharmacists per 10 000 population [31] which is quite high compared with the 2017 median global density of pharmacists to population and even higher than the average pharmacist-to-population ratio reported by OECD Health report in 2017 (8.2 pharmacists per 10 000 population). The experience of other countries shows that this may enhance the risk of unemployment among pharmacy graduates or it may force pharmacists’ income down [32]. Hence, the newer CPs higher interest in embracing expanded scope of practice and their tolerance of more pressure concerns could be for the sake of obtaining and maintaining professional employment.

Similarly, CPs having extensive working hours, and working in pharmacies that are operating round-the-clock were more willing to be involved in influenza vaccination. CPs are already recognized for their convenient locations with more accessible opening times than traditional immunizers, and these have been identified as reasons for patients choosing to attend pharmacy rather than their family doctor for their flu vaccination [33].

Additionally, CPs showing positive attitude towards immunization and towards benefits of provision of influenza vaccines were more likely to be influenza immunizer. Our results are consistent with a study conducted in Canada regarding attitudes and beliefs, where 82% of the Canadian health care providers have a positive attitude toward the expansion of the pharmacists' scope of practice to include provision of vaccines to adults [34].

On the contrary, CPs who perceived patient privacy as barrier had a lower willingness to administer influenza vaccine. This finding is consistent with the result of a study conducted in Lebanon which displayed that lack of privacy constitutes a barrier for patients and this was due to the absence of privacy area inside the pharmacies [22]. However, this might be a major barrier for patient to refer to the CP for seeking immunization service. Hence, we suggest that Lebanese CPs take this aspect into consideration.

Another concern perceived by CPs that affect negatively their intention to administer influenza vaccine was related to the time and cost associated with professional development of CPs. This could be addressed through the designation of an accredited public institution or academy or third party provider to deliver such efficient and recognized courses and training that meets requirements specified by OPL, MOPH and other stakeholders, in affordable prices during short duration of time. A success story was reported in Portugal where using a standardized approach and rolling out of 3 months training in a short space of time, allowed 1273 Portuguese pharmacists (48% of pharmacies) across the country completed the training [35]. Additionally, CPs who considered that patients have a trust issue towards them were less likely to incorporate such service in their settings. In a study assessing patient perspectives toward pharmacy practices in Lebanon, the relevant identified aspects were respect, empathy, friendly staff, listening carefully, giving quality time, responding quickly to their needs and respecting their privacy. Focusing on those aspects through a multifaceted approach is required to enhance pharmacy image and build a longtime confident relationship to encourage patients to come to pharmacy for their flu vaccination [11]. Extensive awareness of the role of CPs as health care provider, strong engagement of CPs in a firm reliable rapport with their customers are among the most important drivers of success of pharmacist-led flu vaccinations. Similarly, remuneration impacted negatively the CP’s intention to vaccinate. However, appropriate remuneration is one condition for effective instigation of vaccinations in pharmacies, which should encourage CPs to provide this kind of service and to further develop pharmaceutical care. Similarly to Lebanon, the costs of influenza vaccinations are borne by patients in Portugal [23]. Without adopting a cost–benefit approach combined to a standardized remuneration among pharmacies, CPs could lose their interest and shift to be reluctant to expand their practice scope.

In summary, community pharmacy as a profession is travelling a complex journey, beginning as a profession concerned with dispensing medicines, and evolving into provision of vaccination services. With limited public health budget, accompanied with a rising in at-risk groups and ailing population, engaging CPs in preventive actions including immunization presented an opportunity to increase influenza vaccination coverage rates and herd immunity, and thus has a significant benefit on public health. In addition, using CPs as immunizer could increase the state of readiness for action in the face of any pandemic, to lower the costs of treatment of infectious diseases and the subsequent complications. Although multiple strategies are required to implement such a service in community pharmacy, including training, marketing, stakeholder engagement and regulatory frameworks, community pharmacy boost the coverage of the target populations and improve patient health.

## Limitations

Several limitations should be acknowledged in the present study. First, our study relies on community pharmacists’ self-reported information, which makes it prone to the disadvantages of desirability biases. Furthermore, this online questionnaire might have favored a selection bias since it might only allow the participation of community pharmacists who have access to online resources to participate. To reduce selection bias related to the use of online surveys, participants were randomly selected from the list of CPs provided by the OPL. Recruited participants were contacted via phone call and were asked to fill in the questionnaire. Thus a high response rate (93%) was reached. However, results may not be generalized to community pharmacists in other countries. Finally, confidence intervals of some adjusted OR were relatively wide. This could be explained by the low sample size in the subcategories.

### Implications

Prior to instigate a community pharmacies-led for influenza vaccination, there is a need to build CPs competencies and skills by establishing well-structured training and under the supervision of competent dons. Simultaneously, it is important to target other healthcare providers to find a framework where they could cooperate with pharmacists in order to reduce any conflict of interest. For future research, it is important to explore community pharmacists’ knowledge and attitudes related to immunization provision. Exploring the perspectives of patients towards receiving influenza vaccines in community pharmacies is recommended.

## Conclusion

Lebanese CPs are willing to provide influenza immunization services. Grasping the unveiled opportunities and overcoming the unearthed concerns related to utilizing CPs as influenza immunizers through a concerted effort is a key to success in any future implementation of vaccination services in pharmacies. Further studies exploring patients’ acceptability to receive such service from CPs are recommended.

## Abbreviations

CPs: Community pharmacists
SPSS: Statistical Package for Social Sciences
CI: Confidence interval
OR: Odds ratio
aOR: Adjusted odds ratio
GAP: Global Action Plan
WHO: World Health Organization
EMR: Eastern Mediterranean Region
OPL: Order of Pharmacists
MOPH: Ministry of Public Health
APhA: American Pharmacists Association
